# Asymmetric saccade reaction times to smooth pursuit

**DOI:** 10.1007/s00221-015-4323-8

**Published:** 2015-06-06

**Authors:** Hans-Joachim Bieg, Lewis L. Chuang, Heinrich H. Bülthoff, Jean-Pierre Bresciani

**Affiliations:** Department of Human Perception, Cognition, and Action, Max Planck Institute for Biological Cybernetics, Tübingen, Germany; Department of Medicine, University of Fribourg, Fribourg, Switzerland; Laboratoire de Psychologie et Neurocognition, CNRS, UMR 5105, Université Pierre Mendes France, Grenoble, France; Department of Brain and Cognitive Engineering, Korea University, Seoul, Korea

**Keywords:** Eye movements, Saccades, Reaction time, Smooth pursuit, Attention, Motion, Inhibition

## Abstract

Before initiating a saccade to a moving target, the brain must take into account the target’s eccentricity as well as its movement direction and speed. We tested how the kinematic characteristics of the target influence the time course of this oculomotor response. Participants performed a step-ramp task in which the target object stepped from a central to an eccentric position and moved at constant velocity either to the fixation position (foveopetal) or further to the periphery (foveofugal). The step size and target speed were varied. Of particular interest were trials that exhibited an initial saccade prior to a smooth pursuit eye movement. Measured saccade reaction times were longer in the foveopetal than in the foveofugal condition. In the foveopetal (but not the foveofugal) condition, the occurrence of an initial saccade, its reaction time as well as the strength of the pre-saccadic pursuit response depended on both the target’s speed and the step size. A common explanation for these results may be found in the neural mechanisms that select between oculomotor response alternatives, i.e., a saccadic or smooth response.

## Introduction

Saccades are rapid eye movements that bring the retinal image of an object to the fovea, the area of highest acuity (Carpenter 1988; Gilchrist 2011). Two processes are thought to control saccades. First, those that determine the endpoint location of the saccade (*where*), and second, processes that determine the timing of the saccade onset (*when*) (Findlay and Walker 1999; Becker and Jürgens 1979).

In saccades to moving objects, both decisions have to be coordinated, since the object’s position continues to change until the saccade is executed. Several studies have investigated saccades to moving objects, in particular, addressing the *where* aspect of saccadic control. These studies have shown that saccades are programmed to compensate for target movements according to the target’s velocity (Keller and Johnsen 1990; Gellman and Carl 1991; Kim et al. 1997; Eggert et al. 2005a, b; Guan et al. 2005; de Brouwer et al. 2002a; Etchells and Benton 2010; but see also Heywood and Churcher 1981; Smeets and Bekkering 2000). For example, in a recent study by Etchells and Benton (2010), participants performed saccades to targets that moved horizontally at varying speeds. Their analysis showed that saccade endpoint error was best explained by a model that incorporates both the target’s position and the velocity of the target 100–300 ms before saccade onset.

The *when* aspect of saccades to moving stimuli has primarily been investigated in the context of smooth pursuit eye movements (reviewed by Ilg 1997; Krauzlis 2005; Thier and Ilg 2005; Barnes 2008). Here, the conditions have been examined that determine the presence or absence of an initial saccade at the beginning of smooth pursuit (Rashbass 1961; Lisberger 1998; Gellman and Carl 1991; de Brouwer et al. 2002a). When following a moving object, its retinal image is first foveated by an initial saccade and then stabilized by a smooth movement of the eye with matching speed (Lisberger 1998). However, a saccade at the beginning of smooth pursuit is not always present. Its occurrence depends on the *zero-crossing* (Gellman and Carl 1991) or *eye-crossing* time (de Brouwer et al. 2002a). For example, smooth pursuit commences directly when the target object crosses the observer’s current fixation location within typical saccade latencies of approximately $200\,\hbox {ms}$ (Rashbass 1961).

Relatively little is known about how the time course of saccade preparation (i.e., saccade reaction times, SRTs) is affected by the properties of a moving target (e.g., the movement direction, speed, or eccentricity). Previous studies suggest that perception of motion may be asymmetric (Mateeff and Hohnsbein 1988; Mateeff et al. 1991; Raymond 1994; Jancke et al. 2004). For example, observers were faster to detect motion onsets of objects that moved toward the fovea (foveopetal) than away from it (foveofugal) (Mateeff et al. 1991). Similar asymmetries could affect the preparation of saccades to moving targets. Indeed, SRTs to moving targets have been shown to depend on the target’s motion direction. However, the reasons for this asymmetry are not clear. Studies by Gellman and Carl (1991) and Moschner et al. (1999) show that saccades to foveopetal targets exhibit longer SRTs than saccades to foveofugal targets. In both studies, target speed and step amplitudes were selected close to zero-crossing times of $200\,\hbox {ms}$. As a result, initial saccades to the moving target were mostly suppressed, and measurements of SRTs were taken from corrective saccades that occurred after the target crossed the zero location. In this case, the reported differences in SRTs may not be related to the target’s motion but directly to saccade cancellation.

A similar study by de Brouwer et al. (2002b) measured the occurrence of saccades in relation to the zero-crossing time. Their results support earlier findings (e.g., Rashbass 1961) by showing that saccades are suppressed if zero-crossing occurs within approximately 40–180 ms. Their data also show an increase in SRTs for saccades in the vicinity of this “smooth zone.” The reasons for this increase remain unclear: de Brouwer et al. (2002b) measured SRTs not from static fixation but during ongoing smooth pursuit. The oculomotor system can quickly respond with an adjustment of smooth pursuit gain to perturbations of the target’s position during smooth pursuit (Carl and Gellman 1987; Morris and Lisberger 1987; Schwartz and Lisberger 1994). Thus, the increase in SRTs in the experiment by de Brouwer et al. (2002b) could either be due to the relative motion of the target or related to the adjustments of smooth pursuit gain, which, when inaccurate, can eventually lead to a correction in the form of a small saccade.

The current study examined how the relative motion direction of the saccade target affected saccade latencies. Importantly, we examined saccades from static fixation and for zero-crossing times greater than the critical suppression value of $200\,\hbox {ms}$. This allowed us to study changes in SRTs without the confounds that were mentioned above. For the first time, the current results demonstrate that saccade latencies depend on the relative motion direction of the saccade target. The implications of this finding for models of saccade preparation and oculomotor response selection are addressed in the “Discussion” section.

## Methods

### Participants

Thirty-two participants took part in the experiment, 16 in the 20°/s condition (8 males, 8 females, age 23–44 years) and 16 in the 10°/s condition (7 males, 9 females, age 19–31 years). All participants had normal or corrected-to-normal vision. In accordance with the Declaration of Helsinki, written informed consent was obtained from all participants prior to the experiment. Participants were paid 8 EUR per hour for taking part in the experiment.

### Materials

Participants sat in an adjustable chair in front of a TFT monitor (Samsung 2233RZ, $120\,\hbox {Hz}$ refresh rate, resolution $1680 \times 1050$, see also Wang and Nikolić 2011). A chinrest provided support for the head at a viewing distance of $57\,\hbox {cm}$. An optical infrared head-mounted eye-tracking system was used to measure gaze at a sampling rate of $500\,\hbox {Hz}$ (SR Research Eyelink II).

### Task

**Fig. 1:**
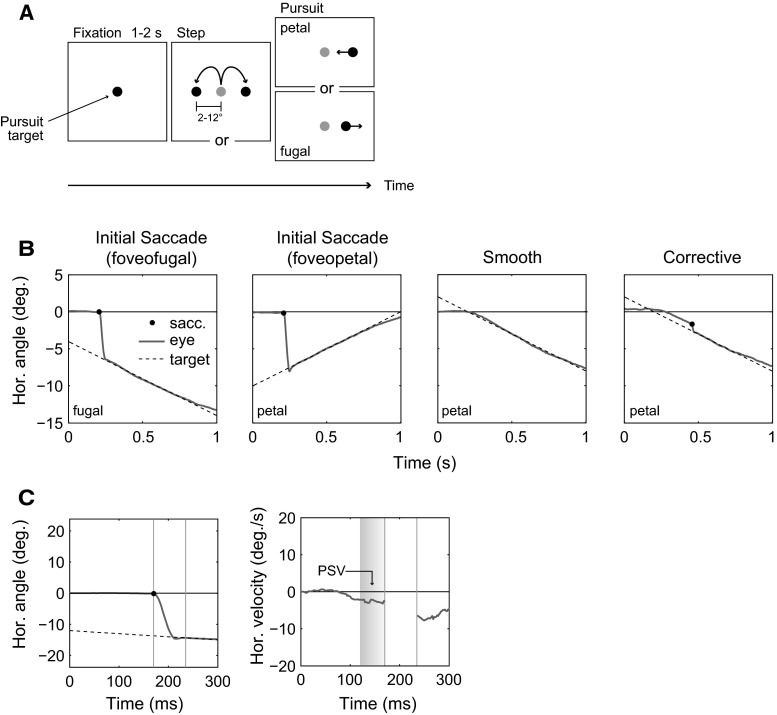
**a** Schematic of the experimental task. Participants fixated a disk at the center of the screen. The disk randomly stepped to the *left* or *right* of the display. After the step, the disk moved at a constant velocity either away from the center (foveofugal) or toward the center (foveopetal). **b** Observed responses. In foveofugal trials, participants always performed a saccade in the direction of motion to catch up with the target. In foveopetal trials, observers either performed a saccade against the motion direction before the target crossed the fixation point or directly initiated a smooth pursuit movement. In some instances, a small corrective saccade occurred after smooth pursuit onset to compensate for inaccurate pursuit (*right graph*). **c** Example position (*left*) and velocity plot (*right*) in a foveofugal trial. A small increase in eye velocity shortly before saccade onset can be measured (pre-saccadic velocity, PSV). The *shaded area* in the velocity plot shows the PSV averaging window (see “Pre-saccadic pursuit” section), the saccade is omitted in this plot

Participants followed the horizontal motion of a pursuit target as closely as possible (see Fig. 1a). The pursuit target was shown on a computer screen and consisted of a disk that was rendered with a smooth, circular gradient from white to gray (RGB 255, 255, 255; to RGB 100, 100, 100) and subtended 0.8°. Throughout the experiment, a uniform gray background (RGB 100, 100, 100) was presented. At the beginning of a trial, the disk appeared at the center of the display. The disk then stepped either to the left or right after a random delay between 1 and 2 s. The amplitude and direction of the step were selected randomly. Six different amplitudes from 2° to 12° (in steps of 2°) were presented. After the step, the disk moved with a constant velocity of 20 or 10°/s either toward the observer’s fixation location (foveopetal) or away from it (foveofugal). One out of 25 trials was randomly designated a catch trial in which no target step occurred.

### Design and procedure

Target speed was varied between subjects. The experiment was run with target speeds of 20°/s for 16 participants and 10°/s for 16 participants. Within-subject factors were the movement direction after the target step (foveopetal, foveofugal) and the target step amplitude (six amplitudes, see above).

In foveopetal trials, this resulted in different zero-crossing times. This is the time that the target requires to reach its original (zero) position after the step. The presented step amplitudes resulted in zero-crossing times from 100 to $600\,\hbox {ms}$ (in steps of 100) for target speeds of 20°/s and 200 to $1200\,\hbox {ms}$ (in steps of 200) for target speeds of 10°/s.

During a session, tasks were presented in several runs. Each run took ca. 15 min including setup and calibration of the eye-tracker. During a run, participants performed five blocks of the experimental task with 25 trials each. Each participant performed four or five runs (in total: 500 and 625 trials, respectively, including catch trials), as conditions permitted. Regular breaks were provided after each run, during which the eye-tracker was removed. The entire experimental session lasted ca. 120 min.

### Data analysis

Saccade detection was carried out by the Eyelink II system using a velocity (22°/s) and acceleration threshold (3800°/s^2^). Pre-saccadic pursuit velocity was calculated using two-point differentiation of the position signal. The velocity signal was averaged for the statistical analysis (“Pre-saccadic pursuit” section), and a low-pass filter ($40\,\hbox {ms}$ symmetrical moving average) was applied for the graphical analysis (Fig. 1c).

The primary measure used was SRT. SRT was defined as the time between the onset of the target step and initiation of the saccade.

Data from the following trials were removed prior to saccade analysis: Trials with blinks during the critical time period shortly before or after the target step missed trials (no saccade or RT greater than $800\,\hbox {ms}$), anticipatory saccades (RT smaller than $80\,\hbox {ms}$, see Wenban-Smith and Findlay 1991), and inaccurate saccades with errors larger than 2° visual angle. Based on this method $5.2\,\%,$ data points were removed in the 20°/s condition and $5\,\%$ in the 10°/s condition. The median number of data points remaining per participant, velocity, step amplitude, and motion direction condition was 40 (min 21).

If not indicated otherwise, data plots show Cousineau–Morey confidence intervals (see Baguley 2012; Morey 2008). Greenhouse–Geisser corrections were applied to ANOVA with more than two-factor levels in case of sphericity violations. Paired, two-tailed *t* tests were employed for post hoc contrast analysis. If not indicated otherwise, linear regression analysis was conducted per participant, and average regression parameters are reported. One-sample, two-tailed *t* tests were employed to assess the significance of the linear relationship.

## Results

### Response types

A typical response to a target step and foveofugal motion is shown in Fig. 1b (left). Here, a saccade is initiated after a latency of approximately $200\,\hbox {ms}$ in the direction of motion of the pursuit target. In trials with foveopetal motion (toward the fovea), several different responses can be observed: (1) an initial saccade before the target crosses the fixation location, (2) direct initiation of smooth pursuit without an initial saccade, or (3) direct initiation of smooth pursuit followed by a small corrective saccade that compensates for inaccuracies in pursuit (Fig. 1b). In the following analysis, smooth responses and responses with a corrective saccade are collapsed (*other* responses). A response is considered to exhibit an initial saccade (*saccade* response) if the saccade occurs before the pursuit target reaches the zero position (central position before the step).

The proportion of responses with initial saccades in the foveopetal condition is expected to depend on the step amplitude and speed of the pursuit target (de Brouwer et al. 2002b). Smooth responses are expected to occur when the target reaches the zero position early, that is, for small step amplitudes and high speeds. Saccadic responses are expected to occur when the target reaches the zero position late, i.e., for large amplitudes and low speeds. To verify this, the proportion of trial types were computed across all subjects and step amplitudes separately for each velocity condition.

**Fig. 2:**
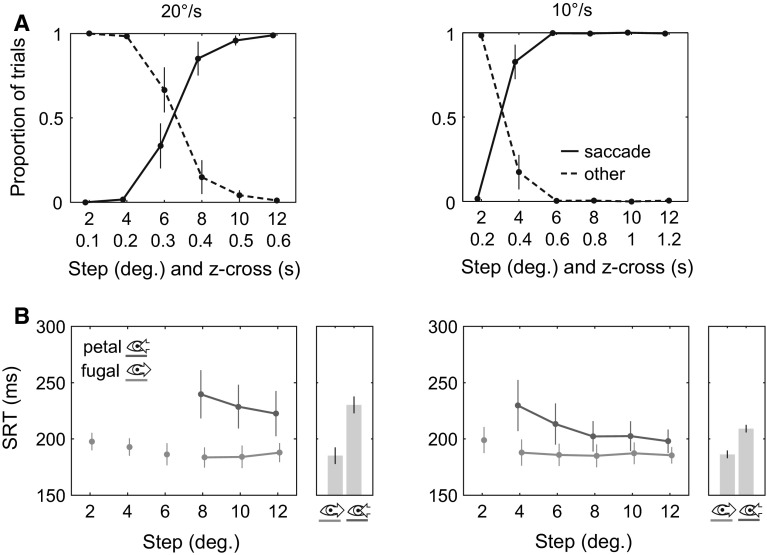
**a** Proportion of response types in foveopetal trials. Responses either exhibited an initial saccade or were entirely smooth or smooth with a corrective saccade (*other* responses). *Error bars* show standard deviations. **b** Average SRT for initial saccades per step and motion condition. Data points for conditions where the proportion of initial saccades in foveopetal trials was <50 % are omitted. Connected data points show conditions that were subjected to an analysis of variance. *Bar charts* to the *right* of each graph show the average SRTs for each motion condition. *Error bars* show standard deviations (*line charts*) and $95\,\%$ Cousineau–Morey CIs (*bar charts*)

Fig. 2a shows histograms of observed responses. The results show that smooth/corrective (other) responses occurred primarily for short step amplitudes and that responses with initial saccades occurred primarily for large step amplitudes. The results show that saccadic responses constituted the majority (>50 %) for steps equal to or larger than 8° in the 20°/s condition and 4° in the 10°/s condition.

### Saccade reaction times

We compared SRTs of initial saccades in foveopetal and foveofugal trials. SRTs were only compared between conditions in which responses with initial saccades constituted the majority in foveopetal trials (see previous section). The RTs of these saccades were expected to be influenced directly by the properties of the target (eccentricity, speed, movement direction) rather than auxiliary factors such as adjustments of pursuit gain or cancellation delays (see “Introduction” section). SRTs of both velocity conditions were analyzed separately because of the different distributions of response types between both conditions. The results are presented in turn.

For trials in the 20°/s condition, a $2\times 3$ repeated-measures ANOVA was conducted. The tested factors include the target motion direction (petal, fugal) and three step amplitudes (8°, 10°, 12°). These were the step amplitudes where the predominant response was an initial saccade in foveopetal trials (>50 %). Figure 2b (left) shows the average SRTs and tested conditions. The analysis shows significantly longer SRTs in saccades to foveopetal targets ($230\,\hbox {ms}$) in comparison with saccades to foveofugal targets [$185\,\hbox {ms}$, $F(1,15)=41.8$, $p<0.01$]. Furthermore, the analysis reveals a significant main effect of step amplitude [$F(2,30)=3.5$, $p<0.05$] and a significant interaction between both factors [$F(2,30)=12.6$, $p<0.01$].

A linear regression of step amplitude and SRT shows a negative relationship for saccades in foveopetal trials [−4.4 ms/°, $t(15)=3.1$, $p<0.01$, average $r^{2}=0.7$] but not for saccades in foveofugal trials. In other words, SRTs decreased with increasing step amplitude for saccades to foveopetal targets but not for saccades to foveofugal targets.

For trials in the 10°/s condition, the range of step amplitudes that were considered in the analysis was extended from 4° to 12° (Fig. 2b, right). Again, these were the step amplitudes where the predominant response was an initial saccade in foveopetal trials. A $2\times 5$ repeated-measures ANOVA shows significantly longer SRTs in saccades to foveopetal targets ($209\,\hbox {ms}$) in comparison with saccades to foveofugal targets [$186\,\hbox {ms}$, $F(1,15)=50.7$, $p<0.01$]. The analysis also shows a significant main effect of step amplitude [$F(4,60)=7.4$, $p<0.01$] and a significant interaction between both factors [$F(4,60)=16.7$, $p<0.01$].

A linear regression of step amplitude and SRT shows a negative relationship for saccades to foveopetal targets (−3.8 ms/°, $t(15)=3.6$, $p<0.01$, average $r^{2}=0.66$) but not for saccades to foveofugal targets.

Post hoc comparisons of both motion directions (petal, fugal) were performed for each step amplitude. This comparison shows significant differences in both speed conditions for the relevant step amplitudes (8°–12° in 20°/s trials, 4°–12° in 10°/s trials, $p<0.01$, Bonferroni corrected).

### Saccade amplitudes

**Fig. 3:**
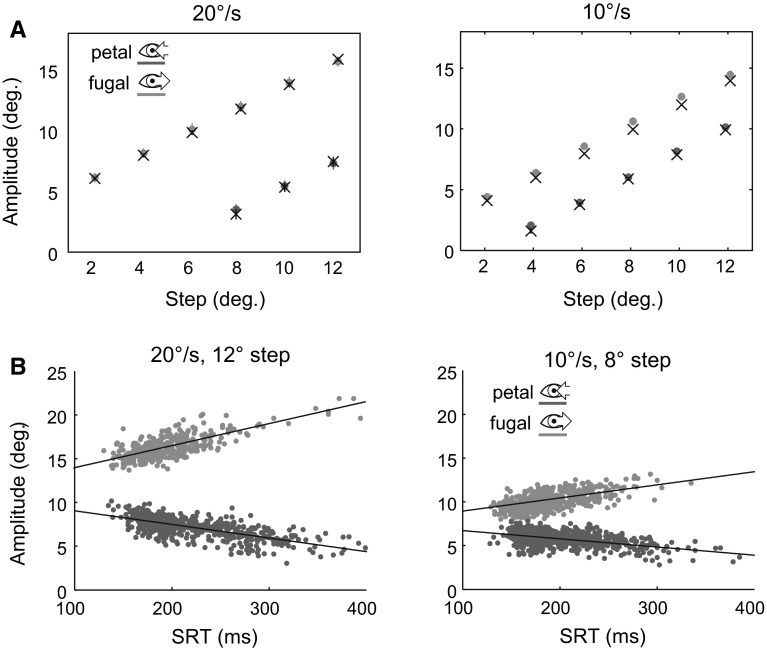
**a** Average saccade amplitude for initial saccades per step and motion condition. *Crosses* show predicted amplitudes based on the initial target step and target motion during the average SRT for the respective motion and step condition. *Error bars* show standard deviations. **b** Linear relationship between SRT and saccade amplitude for two representative motion and step conditions. *Data points* show individual trials for all participants, and *lines* show best linear fit. The distribution of saccade amplitudes shows an adjustment to match the retinal error that results from the target’s motion during the saccade preparation period, i.e., they become larger in the foveofugal condition since the target moves away from the current fixation position and smaller in the foveopetal case, since the target moves toward the fixation position

Previous research has shown that the displacement of the target during the saccade preparation period is taken into account by the saccade planning process (Guan et al. 2005): For example, after the initial step, as the target travels further into the periphery during foveofugal trials, saccade amplitudes become larger. The current data show that saccade amplitudes are predicted by the target displacement of the initial step and the target’s motion during the SRT (see also Fig. 3a): For trials in the 20°/s condition, the average difference between predicted and actual amplitude was 0.08°. For trials in the 10°/s condition, the average difference between predicted and actual amplitude was 0.03°.

A linear regression analysis of saccade amplitude and SRT was conducted to examine the relationship between saccade amplitude and SRT in greater detail (see also Fig. 3b). Linear regression slopes were computed per participant and condition. Repeated-measures ANOVA was employed to analyze the slope parameters (same conditions as in “Saccade reaction times” section). In line with previous results (Guan et al. 2005), this analysis shows that saccade amplitudes incorporate the displacement of the target during the saccade preparation period: For trials in the 20°/s condition, the analysis shows a negative relationship for saccades in foveopetal trials [−16°/s, $F(1,15)=240$, $p<0.01$] and a positive relationship for saccades in foveofugal trials (26°/s). For trials in the 10°/s condition, the analysis also shows a negative relationship for saccades in foveopetal trials [−7°/s, $F(1,15)=367$, $p<0.01$] and a positive relationship for saccades in foveofugal trials (15°/s). For both target speed conditions, the results neither showed a main effect of step amplitude nor an interaction between motion direction and step amplitude ($p>0.1$).

### Eccentricity-matched SRTs

The differences in saccade amplitudes between both motion direction conditions suggest one potential explanation for the obtained SRT results, namely differences in the eccentricities of targets prior to saccade onset. Previous work on saccades to static targets has shown that target eccentricity affects SRTs (Kalesnykas and Hallett 1994). The results presented in the previous section demonstrate that saccade amplitudes vary as a function of time, in line with a change in eccentricity due to the target’s constant movement. For example, the eccentricity of a target shortly before the saccade becomes larger if the target moves foveofugally and smaller if it moves foveopetally. To test whether the obtained differences in SRTs of initial saccades were independent from the differences in target eccentricity during the saccade preparation period, a comparison of eccentricity-matched conditions was conducted. To select matching step amplitude conditions from the available conditions of our experiment, we assumed a baseline saccade latency of $200\,\hbox {ms}$. This is a typical average saccade latency (Kalesnykas and Hallett 1994). For example, in the current experiment, target eccentricity following a 12° step and foveopetal motion was approximately 8° after $200\,\hbox {ms}$ of motion at a speed of 20°/s. This eccentricity condition was matched by a 4° step and foveofugal motion at the same speed.

**Fig. 4:**
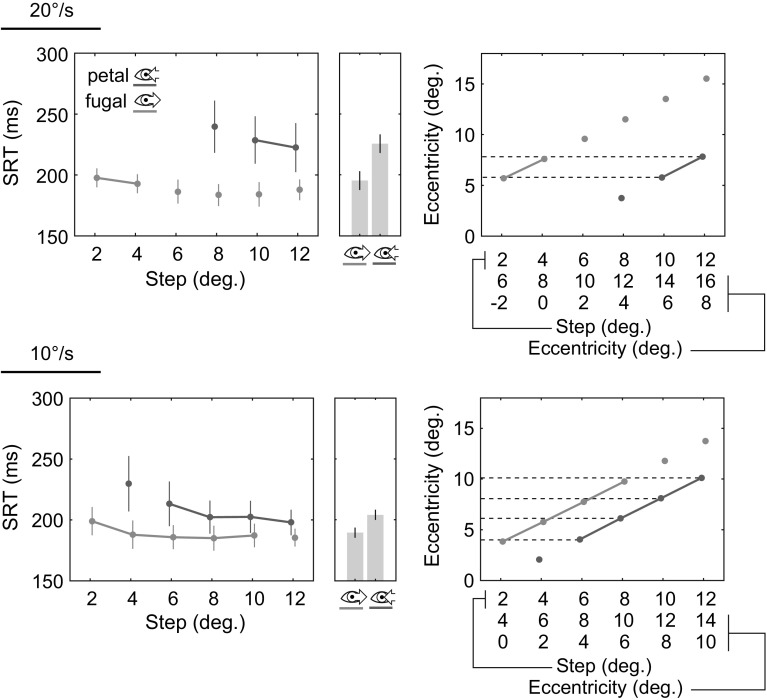
Eccentricity-matched motion conditions. *Top* 20°/s trials. *Bottom* 10°/s trials. *Left* SRTs of the conditions that were compared in the statistical analysis are connected. *Bar charts* to the *right* of each graph show the average SRTs for each motion condition for the compared conditions. *Right* actual eccentricities of targets shortly before saccade onset of the compared conditions. Eccentricities in the foveopetal and foveofugal case were approximately equal (see text for details). X-axis labels show the predicted eccentricity of the target after the step and $200\,\hbox {ms}$ of motion for each motion and step amplitude condition

It should be noted that these predicted eccentricities approximate the actual target eccentricities, for example, due to differences in actual SRTs. In contrast to the unmatched comparison, eccentricities in both motion conditions are now reversed in the matched comparison: on average slightly larger in the foveopetal motion condition and slightly smaller in the foveofugal condition (see Fig. 4a). If target eccentricity is the main determinant of saccade latencies, saccades to foveopetal targets should exhibit shorter rather than longer SRTs in this matched comparison. The results show that this is not the case: For 20°/s trials, the conditions selected for comparison were 2° and 4° steps for foveofugal trials and 10° and 12° steps for foveopetal trials. The targets in these two conditions were approximately at 6° and 8° eccentricity at saccade onset, respectively (Fig. 4a). A $2\times 2$ repeated-measures ANOVA shows significantly longer SRTs in saccades to foveopetal targets ($225\,\hbox {ms}$) in comparison with saccades to foveofugal targets [$195\,\hbox {ms}$, $F(1,15)=17.9$, $p<0.01$].

For 10°/s trials, the selected conditions were 2°, 4°, 6°, and 8° steps for foveofugal trials and 6°, 8°, 10°, and 12° steps for foveopetal trials. The targets in these conditions were approximately at 4°, 6°, 8°, and 10° eccentricity at saccade onset, respectively (Fig. 4a). Again, a $2\times 4$ repeated-measures ANOVA shows significantly longer SRTs in saccades to foveopetal targets ($209\,\hbox {ms}$) in comparison with saccades to foveofugal targets [$186\,\hbox {ms}$, $F(1,15)=50.7$, $p<0.01$].

Post hoc comparisons of both motion directions (petal, fugal) were performed for each matched step amplitude, showing significant differences in both speed conditions for the relevant step amplitudes (6° and 8° in 20°/s trials, 4°–10° in 10°/s trials, $p<0.05$, Bonferroni corrected).

Together, this suggests that the measured SRT differences are a result of the difference in the target’s motion direction rather than differences in its eccentricity prior to saccade onset.

### Pre-saccadic pursuit

Small, pre-saccadic movements in the direction of pursuit can sometimes be observed in saccades to moving targets (Tychsen and Lisberger 1986). The occurrence of these eye movements could be related to saccade onsets. Saccades to foveopetal targets are made against the motion of occurring pre-saccadic pursuit. This means that the eyes are first decelerated before they are accelerated in the opposite direction. This could potentially affect the measurements of saccade onsets based on velocity and acceleration thresholds and thus lead to prolonged SRT measurements.

To examine the occurrence and relationship of these eye movements with the measured SRTs, we computed the average eye velocity (pre-saccadic velocity, PSV) $50\,\hbox {ms}$ prior to the saccadic eye movements (see also Guan et al. 2005). Statistical analyses were performed for conditions in which the predominant response was an initial saccade in foveopetal trials. PSV values are reported relative to the pursuit target’s velocity, i.e., positive values indicate that pre-saccadic pursuit moved the eye in the target’s motion direction.

For trials in the 20°/s condition, a $$2\times 3$$ repeated-measures ANOVA was conducted. The tested factors include the target motion direction (petal, fugal) and three step amplitudes (8°, 10°, 12°, the same as in “Saccade reaction times” section). In both motion conditions, PSVs occurred in the direction of target motion. For foveopetal movements, PSVs were faster (2.4°/s) than for foveofugal movements [0.7°/s, $$F(1,15)$$$$=18.0$$, $$p<0.01$$]. Furthermore, the analysis reveals a significant main effect of step amplitude [$$F(2,30)=12.1$$, $$p<0.01$$] and a significant interaction between both factors [$$F(2,30)=16.3$$, $$p<0.01$$].

For trials in the 10°/s condition, a $2\times 5$ repeated-measures ANOVA was conducted. The range of step amplitudes that were considered in the analysis was extended from 4° to 12° (see “Saccade reaction times” section). For this target speed, PSVs did not differ significantly between both conditions (on average 0.81°/s). The analysis shows a significant main effect of step amplitude [$F(4,60)=25.1$, $p<0.01$] and a significant interaction between both factors [$F(4,60)=14.7$, $p<0.01$].

Linear regression of step amplitude and PSV shows that, similar to the SRT results, PSVs decreased with increasing step amplitude for foveopetal target motion [20°/s motion: −0.4°/s per degree, $t(15)=4.3$, $p<0.01$, average $r^{2}=0.67$; 10°/s motion: −0.17°/s per degree, $t(15)=5.1$, $p<0.01$, average $r^{2}=0.68$].

A detailed analysis of the relationship between SRTs and PSVs was conducted. First, linear regression slopes were computed per participant and condition. Next, for trials in the 20°/s condition, a $2\times 3$ repeated-measures ANOVA was conducted with the slope parameter as dependent variable (for factor levels, see “Saccade reaction times” section). In both motion conditions, PSVs increased slightly during saccade preparation. For foveofugal movements, PSVs increased by 0.75° per $100\,\hbox {ms}$ and by 3.7° per $100\,\hbox {ms}$ for foveopetal movements [$F(1,15)=50.2, p<0.01$]. The analysis also shows a significant interaction between the step and motion direction factors [$F(4,60)=14.0$, $p<0.01$].

For trials in the 10°/s condition, a $2\times 5$ repeated-measures ANOVA was conducted with the slope parameter as dependent variable. Again, PSVs increased slightly during saccade preparation in both motion conditions. For foveofugal movements, PSVs increased by 0.65° per $100\,\hbox {ms}$ and by 1.7° per $100\,\hbox {ms}$ for foveopetal movements [$F(1,15) =52.5, p<0.01$]. The analysis also shows a significant interaction between the step and motion direction factors [$F(4,60)=6.5$, $p<0.01$].

Linear regressions of step amplitude and slope parameters confirm the general results: As step amplitudes increased, slopes became more shallower for foveopetal target motion [20°/s motion: −0.42° per $100\,\hbox {ms}$ per degree, $t(15)=4.0$, $p<0.01$, average $r^{2}=0.32$; 10°/s motion: −0.21°/s per degree, $t(15)=5.6$, $p<0.01$, average $r^{2}=0.48$].

These results can be explained by assuming that PSVs (as SRTs) are affected by the target’s eccentricity and speed (e.g., Tychsen and Lisberger 1986) but do not elucidate the causal relationship between both.

A subset of trials was analyzed to examine whether the measured SRT asymmetries depend on the occurrence of PSVs. The subset comprises of trials with absolute PSVs smaller than 1°/s, i.e., trials wherein the eye fixation was relatively static (Guan et al. 2005). This analysis was only performed for 10°/s trials with target step amplitudes larger or equal to 6°, to assure that data points were available for the analysis for all participants and conditions ($70\,\%$ of datapoints).

The average PSV in this subset was 0.07°/s. A $2\times 4$ repeated-measures ANOVA shows significant differences in SRTs between both motion conditions also in this subset. SRTs were longer in saccades to foveopetal targets ($198\,\hbox {ms}$) in comparison with saccades to foveofugal targets [$185\,\hbox {ms}$, $F(1,15)=31.3$, $p<0.01$].

Post hoc comparisons of both motion directions (petal, fugal) were performed for each step amplitude of the subset. This shows significantly longer SRTs for saccades to foveopetal targets for the relevant step amplitudes (6°–12°, $p<0.05$, Bonferroni corrected).

These results suggest that, although pre-saccadic responses appear to be similarly affected by the target’s motion and eccentricity as SRTs, they do not necessarily cause the measured SRT effects.

## Discussion

The current study examined SRT differences to moving targets. The results show that the motion direction of the saccade target influences the saccade’s reaction time: SRTs are longer for targets that move toward the fovea (foveopetal) and shorter for targets that move away from the fovea (foveofugal). We believe that this asymmetry in SRTs is related to the neuronal mechanism of the oculomotor system that determines the appropriate response to fixate a moving target: saccadic or smooth.

### SRT asymmetries

Previous studies reported asymmetries in SRTs when saccades moved the eye *away* from a moving stimulus, i.e., during ongoing smooth pursuit (Tanaka et al. 1998; Blohm et al. 2005; Khan et al. 2010; Bieg et al. 2013). In these studies, SRTs were shorter when the saccade’s direction and the pursuit movement direction matched. This phenomenon has been linked to a bias of visual–spatial attention in the visual hemifield where pursuit is directed (e.g., Khan et al. 2010, see also Donkelaar 1999; Seya and Mori 2012; Sheliga et al. 1994; Deubel and Schneider 1996; de’Sperati and Deubel 2006; Souto and Kerzel 2011; Kerzel et al. 2008, but also Lovejoy et al. 2009). A similar bias in attention, which may result in shorter or longer SRTs, cannot be precluded as an explanation for the current results. However, in the current experiment, smooth pursuit of the target occurred *after* the saccade to the target. The present results may consequently be related to the perception of the target’s motion in the periphery rather than ongoing smooth pursuit.

Evidence for a bias in motion perception has indeed been found by previous research. For example, observers were faster to detect foveopetal motion onsets (see Mateeff et al. 1991 but also see Ball and Sekuler 1980; Naito et al. 2010; Bieg et al. 2013). Such a reduction in motion onset detection time may speak in favor of a general motion processing bias, namely an enhancement of foveopetal motion processing. In contrast, the current results seemingly demonstrate the opposite: Saccades were initiated earlier to foveofugal motion. A motion processing bias, as proposed by Mateeff et al. (1991), may therefore not be directly causing these observations.

Next, we will propose an explanation for the observed results, in the course of which we will return to the role of a general motion processing bias (“Neurophysiological mechanisms” section). We propose that saccadic asymmetries to moving targets are related to the decision between a saccadic or smooth response, specifically the decision to suppress a saccade in favor of a completely smooth response.

The typical oculomotor behavior following a step-ramp involves pre-saccadic pursuit with a short onset time of ca. $100\,\hbox {ms}$, an initial saccade with a typical latency of ca. $200\,\hbox {ms}$, and finally, a phase with stable pursuit (Carl and Gellman 1987). It is useful to suppress a saccade when the object moves toward the fovea, for instance in a foveopetal step-ramp task (Rashbass 1961). In such a task, a target steps from a foveal to a peripheral position before moving in the opposite direction, toward the fovea. The decision to suppress a saccade depends both on the target’s eccentricity and speed. Specifically, the time that is required by the target to again reach its foveal position, the *zero-crossing* or *eye-crossing* time (Gellman and Carl 1991; de Brouwer et al. 2002a), can be used to predict whether a saccade occurs before smooth pursuit commences. For zero-crossing times of approximately $200\,\hbox {ms}$, the initial saccade to the target is suppressed and smooth pursuit of the target begins directly (Rashbass 1961).

It is still debated how the oculomotor system implements this decision. For example, saccades could simply be suppressed once the retinal representation of the target reaches a fixed foveal threshold (Grossberg et al. 2012). Instead, suppression may occur more gradually through influences from motion processing on the saccade preparation process. Prolonged SRTs could speak in favor of this hypothesis (Boucher et al. 2007; Hanes and Schall 1996; Dorris and Munoz 1998). Earlier studies have, in fact, reported prolonged SRTs in saccades to targets that moved foveopetally (Gellman and Carl 1991; Moschner et al. 1999; de Brouwer et al. 2002a). Yet, these results may not be related to the target’s motion itself but to auxiliary phenomena that are a consequence of that motion (e.g., cancellation due to zero-crossing or adjustments of smooth pursuit gain; see “Introduction” section). Recently, we have presented first evidence for a *direct* influence of target motion on saccade preparation (Bieg et al. 2013). In this study, observers were required to alternate their foveal gaze between a moving and static target. For saccades from the static to the moving target, our results showed longer SRTs when the target moved toward the fixated location. Critically, these saccades moved the eyes from a static position, against the motion direction, and occurred before zero-crossing. Therefore, the difference in SRTs appears to be primarily related to the relative motion of the pursuit target.

The current results corroborate and extend this finding. In particular, measurements of SRTs in different step amplitude conditions enabled a comparison of SRTs in eccentricity-matched conditions. Unlike previous work, this allowed us to determine whether the SRT differences between the two motion conditions could be explained solely on the basis of different eccentricities of the target during saccade preparation (Kalesnykas and Hallett 1994, but see also Hodgson 2002; Dafoe et al. 2007). The comparison of SRTs in eccentricity-matched conditions supports our main results. This suggests that SRTs to moving targets may not primarily depend on the target’s eccentricity but also on the relative motion direction of the saccade target.

### Pre-saccadic pursuit

SRT asymmetries could be related to asymmetries in smooth pursuit initiation, specifically the pursuit response that typically occurs before the first saccade to the target (Lisberger and Westbrook 1985; Tychsen and Lisberger 1986; Carl and Gellman 1987). In fact, pre-saccadic pursuit and SRTs appear to exhibit a similar dependency on both the target’s eccentricity and speed. For example, the current and earlier work (Lisberger and Westbrook 1985; Tychsen and Lisberger 1986) have shown stronger pre-saccadic pursuit for targets that move close to the fovea and toward it.

Pre-saccadic responses typically move the eye *against* the upcoming saccade’s motion direction in foveopetal trials. This suggests that prolonged SRTs in saccades against the target’s motion direction may arise from the contrasting momentum of early pursuit and the subsequent saccadic eye movement. Our analysis provides evidence against this assumption: Asymmetries in SRTs also exist in a subset of data without significant pre-saccadic pursuit activity. This suggests that early pursuit behavior may not be a precondition for the occurrence of SRT asymmetries, but the fact that both behaviors appear to be similarly affected by target eccentricity and speed may point to a common underlying influence.

### Neurophysiological mechanisms

This section highlights a possible neuronal explanation of the three phenomena that depend on the target’s relative motion direction: suppression of initial saccades, increase in SRTs, and modulation of pre-saccadic pursuit. A model by Grossberg et al. (2012) explains suppression of initial saccades prior to smooth pursuit as a result of connections between motion-sensitive cortical areas (area MT, i.e., middle temporal area) and saccade-related regions in the superior colliculus (SC), specifically excitatory input to rostral parts of the SC. Neurons in rostral SC are known to activate omnipause neurons, a class of neurons in the brainstem, whose activity suppresses saccades (Munoz and Wurtz 1993; Hafed et al. 2009). Critically, the model by Grossberg et al. (2012) assumes stronger activation of rostral SC for targets that fall near the foveal area in the MT topography. This arrangement effectively implements a foveal threshold that can explain the suppression of a saccade during a foveopetal step-ramp.

The asymmetries in SRTs in the current experiment speak against such a simple threshold model and in favor of a direct and gradual modulation of the saccadic drive signal. This signal is implemented by a second class of neurons in caudal parts of the SC, which are active before and during saccades and whose activity correlates with SRTs (Dorris et al. 1997; Dorris and Munoz 1998; Paré and Hanes 2003). Intracollicular signals from fixation to movement neurons provide inhibitory input and are thereby able to delay the buildup of activity that is necessary to trigger a saccade (Munoz and Istvan 1998; Paré and Hanes 2003). Apart from the suppression of saccades through omnipause neurons, the corticotectal connection suggested by Grossberg et al. (2012) could thus engage a second suppression mechanism that directly affects saccade preparation. Prolonged SRTs, as in the current experiment, may then be regarded as a concomitant of this mechanism, for example in situations in which inhibition of movement-related activity did not suppress saccade initiation completely but only retarded it.

Local inhibitory connections within the SC can explain modulations of SRTs but not the observed asymmetries. This phenomenon may be related to asymmetries that originate directly in motion-sensitive areas such as MT. For example, pre-saccadic pursuit activity is thought to directly reflect the output of these motion processing areas (Newsome et al. 1985; Lisberger and Westbrook 1985; Tychsen and Lisberger 1986; Lisberger and Movshon 1999; see also reviews by Spering and Montagnini 2011; Schütz et al. 2011) and exhibits similar asymmetries (see current results and also Tychsen and Lisberger 1986 Figs. 2a, 4a). Further support comes from psychophysical studies on motion perception that showed earlier detection of foveopetal motion (Mateeff and Hohnsbein 1988; Mateeff et al. 1991; Raymond 1994; Jancke et al. 2004) and brain imaging studies that showed higher activation of MT during foveopetal motion (Naito et al. 2000, 2010). Along the lines of the model proposed by Grossberg et al. (2012), stronger MT signals subsequently lead to greater SC fixation neuron activity and thus a depression of saccade-related activity in caudal SC. This depression can lead to prolonged saccadic latencies, in correspondence with the current results.

### Conclusion

The current study showed that SRTs to moving targets are asymmetric: SRTs to targets that move toward the fovea are longer than SRTs to targets that move away from the fovea. In addition, SRTs to targets that move toward the fovea depend on both the target’s retinal eccentricity and speed. A similar dependency was found for the occurrence of initial saccades in general and for the strength of the pursuit activity that occurred shortly before the saccade to the target. We hypothesize that these phenomena are linked to the mechanisms by which the brain decides on the most appropriate oculomotor response, e.g., saccadic or smooth. The modulation of SRTs could be regarded as a by-product of this decision mechanism.

Lastly, it should be mentioned that the noted asymmetries may also exhibit behavioral advantages: Targets that move into the periphery potentially move out of the visual field. A saccade that is initiated quickly enough can prevent the observer from losing track of the target. In targets that move toward the fovea, a saccade, and the loss of vision that is associated with it, is avoidable. Potentially, the decision to suppress a saccade also benefits from an extended sampling period, which could lead to a more accurate estimate of the target’s velocity (Bruyn and Orban 1988; Bennett et al. 2007, 2010).
